# Machine learning-based analysis of key factors and mediating mechanisms of career decision-making difficulties among higher vocational college students

**DOI:** 10.3389/fpsyg.2026.1871065

**Published:** 2026-09-03

**Authors:** Li Zhang, Huadi Wang

**Affiliations:** 1 School of Education Science, Nanjing Normal University, Nanjing, Jiangsu, China; 2 Student Affairs Office, Jiangsu Vocational Institute of Commerce, Nanjing, Jiangsu, China; 3 Institute of Vocational Education and Lifelong Education, Jiangsu Academy of Educational Sciences, Nanjing, Jiangsu, China

**Keywords:** career decision-making difficulties, decision tree, K-means clustering, random forest, vocational college students

## Abstract

This research explores the mechanisms through which multidimensional predictors shape career decision-making difficulties among vocational college students and identifies disparities in career decision-making difficulties across student subgroups with disparate developmental profiles. Survey participants were recruited from vocational institutions in Jiangsu, Anhui, and Guangxi provinces, with nine predictive constructs selected as research variables: sense of place, parental career-related behaviors, psychological resilience, core self-evaluations, vocational self-concept, meaning in life, career adaptability, future time perspective, and career exploration. The study first employs k-means cluster analysis to categorize student subgroups, followed by random forest modelling to pinpoint pivotal variables contributing to subgroup segmentation. Empirical findings unfold in three core aspects. First, sampled vocational students fall into three distinct clusters defined by developmental resources: high-resource, moderate-resource, and low-resource cohorts based on the nine measured variables. Second, measurable graded discrepancies in career decision-making difficulties emerge across the three subgroups. Third, future time perspective, core self-evaluations, career exploration and psychological resilience stand as dominant predictors differentiating student clusters; specifically, future time perspective negatively forecasts career decision-making difficulties via a serial mediating path sequentially through core self-evaluations, psychological resilience and career exploration. By unpacking subgroup heterogeneity of vocational students’ career decision-making difficulties from an integrated multi-factor framework, this study furnishes empirical evidence and practical implications for vocational colleges to design targeted career counselling interventions and mitigate students’ struggles in career decision-making.

## 1 Introduction

Against the backdrop of high-quality advancement in contemporary vocational education alongside structural shifts across the employment market, vocational college students’ capacity for career decision-making and lifelong developmental trajectories exert direct bearings on their employment outcomes, occupational adjustment and the cultivation of skilled technical workforce. Defined as choices relevant to academic pursuits and occupational pathways made across disparate life stages, career decision-making constitutes a core developmental task throughout individual lifespan (Chi et al., 2016). Characterized by prevalence among vocational undergraduates, career decision-making difficulties refer to the inability to arrive at sound, personalised vocational choices stemming from insufficient occupational information, poor self-awareness or conflicting contextual conditions, ranking among the most prevalent developmental hurdles for this student cohort. Scholarly inquiries into career decision-making difficulties have evolved from unidimensional descriptive documentation toward multidimensional mechanistic exploration. Gati’s three-factor framework categorises career decision-making difficulties into three core components: inadequate preparatory readiness, insufficient informational access and contradictory external information (Gati et al., 1996). Subsequent researchers extended this theoretical framework by incorporating external contextual constraints tailored to vocational learners, encompassing parental involvement and regional employment restrictions. Rather than stemming from isolated contributors, vocational students’ career decision-making difficulties arises from intertwined predictors spanning external surroundings, familial backing, personal psychological dispositions and career-related competencies. Sense of place captures learners’ emotional attachment, regional identification and forward-looking developmental expectations concerning their residing locales, functioning as a pivotal contextual predictor of their occupational preferences and willingness to secure local jobs. Encompassing emotional backing, informational provision and behavioral intrusion, parental career-linked practices serve as the most immediate familial external predictor shaping students’ vocational choices. Constructs including psychological resilience, core self-evaluations and meaning in life represent inherent psychological assets that govern learners’ confidence, emotional stability and perseverance amid decision-related stress. By contrast, career adaptability, future time perspective, career exploration and vocational self-concept jointly govern learners’ proficiency in gathering labor-market intelligence, clarifying self-positioning, drafting developmental roadmaps and reaching appropriate occupational resolutions. Existing empirical literature predominantly examines isolated or a small subset of predictive variables in separation, with scarce scholarship unpacking between-group disparities in career decision-making difficulties from a typological grouping perspective based on varied multidimensional developmental attributes. Driven by growing implementation of artificial intelligence within educational grouping research, analytical tools such as k-means clustering and decision trees have proven advantageous for multidimensional dataset segmentation, paving innovative avenues for granular empirical research on heterogeneous subgroups troubled by career decision-making difficulties.

And most existing literature on career decision-making difficulties initially proposed hypotheses that certain variables will affect career decision-making difficulties, and then verified these hypotheses through traditional analytical methods such as latent profile analysis. Traditional statistical methods, such as regression analysis, typically presuppose linear relationships between variables, making it difficult to capture complex interactions in the real world. In contrast, machine learning algorithms, such as random forests and neural networks, do not rely on such presuppositions and are capable of automatically learning from data and fitting these complex nonlinear patterns. Machine learning, capable of deeply exploring the internal relationships between variables without requiring prior knowledge or experience, is a data-based analysis method. This article first identifies the key factors affecting career decision-making difficulties through machine learning, and then analyzes the mediating chain effect between these identified key factors and career decision-making difficulties, thereby enhancing the credibility and accuracy of career decision-making difficulties analysis.

## 2 Theoretical foundations and literature review

### 2.1 Core theories of career decision-making difficulties

Research on career decision-making difficulties draws on multidisciplinary theoretical frameworks, with scholars continuously refining its conceptual connotation from diverse disciplinary lenses. Classic career decision-making theory, Social Cognitive Career Theory (SCCT), and Career Construction Theory (CCT) elaborate the formative mechanisms of career decision-making difficulties from distinct dimensions, laying theoretical groundwork for dimensional classification and cause analysis of career decision-making difficulties among vocational college students.

#### 2.1.1 Permission to reuse and copyright

Traditional career decision-making theory centers on person-job fit, dating back to Parsons’ (1909) theory of person-job fit. This theory posits that career decision-making essentially involves systematic matching between individual traits and occupational requirements. Failure to clearly perceive personal traits or effectively obtain occupational information directly leads to decision-making difficulties. Holland’s (1959) theory of vocational personality types further elaborates this logic, proposing a matching model of six vocational personality and environmental types (realistic, investigative, artistic, etc.). He clearly states that individuals who cannot identify their personality types or understand occupational environmental characteristics are prone to “type-confusion” decision-making difficulties, providing a theoretical basis for subsequent research on “self-cognition ambiguity” decision-making difficulties among vocational college students. Building on classic theories, Gati et al. (1996) classified career decision-making difficulties into three dimensions: lack of readiness, lack of information, and inconsistent information. This highly operable framework has become a core tool for empirical research and is widely used to identify types of decision-making difficulties across groups.

#### 2.1.2 Social cognitive career theory (SCCT)

Proposed by Lent and Brown (2013, 2020), Social Cognitive Career Theory (SCCT) moves beyond the traditional single focus on matching, taking self-efficacy as the core variable linking individuals and environments, and emphasizing that career decision-making difficulties are closely related to career decision-making self-efficacy. This theory holds that individuals’ beliefs in their ability to complete career decision-making tasks directly influence the initiation and persistence of decision-making behaviors; low self-efficacy leads individuals to avoid decision-making exploration.

The explanatory power of SCCT has been fully verified among vocational college students. Empirical research by Wang et al. (2023) within the SCCT framework shows that vocational college students’ career decision-making self-efficacy not only directly negatively predicts decision-making difficulties but also mediates the relationships between career values and employability (Wang, 2022), as well as between major satisfaction and decision-making difficulties (Hu et al., 2024). Zhong (2023) further found that achievement goals can significantly reduce decision-making difficulties by enhancing career decision-making self-efficacy, indicating that improving self-efficacy is a key pathway to alleviating decision-making difficulties and providing empirical support for the application of SCCT in vocational education.

#### 2.1.3 Career construction theory (CCT)

Career Construction Theory (CCT) was proposed by Savickas (2013), which regards career decision-making as a dynamic process in which individuals actively “construct career meaning” and identifies career adaptability as the core factor coping with decision-making difficulties. This theory defines career adaptability as four dimensions: concern, control, curiosity, and confidence. Individuals with higher adaptability can proactively explore themselves and the environment, flexibly adjust decision-making strategies, and thus reduce decision-making difficulties.

Researchers have found that vocational college students’ major self-concept can significantly reduce decision-making difficulties by improving career adaptability (Hu et al., 2017); major satisfaction exerts a negative predictive effect on decision-making difficulties through the mediating role of psychological resilience (Hu, 2023); and vocational college students with high career adaptability can better cope with external challenges such as parental intervention and information deficiency (Yang et al., 2021). The above empirical studies on vocational college students further validate the explanatory power and value of CCT for career decision-making difficulties in this population.

Prior scholars have explored the intrinsic correlations among psychological resilience, core self-evaluations, career exploration, future time perspective and career decision-making difficulties. He et al. (2024) verified the mediating role of psychological resilience linking the impostor phenomenon to career indecision based on a sample of nursing interns. Using undergraduate students majoring in physical education as research subjects, Wang et al. (2026) confirmed that psychological resilience acts as a mediator between core self-evaluations and career indecision. Chang et al. (2026) further examined the predictive effects of general self-efficacy and psychological resilience on career indecision. Alkal (2025) identified career decision self-efficacy as a mediator in the longitudinal association between psychological resilience and career adaptability. Zhao (2024) investigated the influencing mechanism of meaning in life on career adaptability among secondary vocational students. Hu and Wang (2023) reported that psychological resilience mediates the relationship between meaning in life and internalizing and externalizing problem behaviors in adolescents, while Lin (2024) explored the predictive effect of meaning in life on career adaptability. Xu et al. (2022) demonstrated that future time perspective exerts both direct effects on career indecision and indirect impacts via mediators such as self-esteem. Sarsıkoğlu (2024) documented that elevated future time perspective effectively alleviates career indecision, and Jia et al. (2022) validated the significant sequential mediating effects of career adaptability and anxiety in the pathway from future time perspective to career indecision. Wang (2021) and Ye (2024) discovered that self-esteem serves as a mediator linking future time perspective to meaning in life, and Cao (2023) explored the correlation between future time perspective and psychological resilience. Wang et al. (2025) analyzed the effects of parental career intervention behaviors on the career decision-making difficulties. Ahmadi et al. (2023) provides a clear framework for understanding how environmental and interpersonal factors support or thwart individuals’ psychological needs and motivation. Incorporating this work could perhaps strengthen the theoretical foundation of the manuscript by offering a more integrated explanation of how contextual resources may influence career adaptability, future time perspective, psychological resilience.

### 2.2 Application of machine learning in career decision-making research

With its technical advantages in multi-source data integration and algorithmic modeling, machine learning has been widely applied in career decision-making. Scholars have conducted extensive empirical research on theoretical framework innovation, model optimization and adaptation, application verification, and expansion, forming a multi-dimensional application system and research outcomes that provide strong support for college students’ scientific career decision-making.

#### 2.2.1 Innovation of career decision-making theoretical framework and algorithm model construction

Xu and Flores (2023) proposed a dual-process theory of career decision-making supported by machine learning, breaking through the limitations of traditional models, deconstructing career decision-making as a four-stage process driven by “intuition-analysis” dual pathways, and constructing a trinity system of “theoretical framework-prediction model-practical guidelines,” offering core theoretical support for the dynamic adjustment of college students’ career decision-making under uncertainty.

To address multi-factor interference in career decision-making, Yang et al. adopted machine learning algorithms including decision trees, artificial neural networks, and support vector machines, integrating students’ academic performance, demographic data, and social media news indices by graduation year, improving model performance through dataset segmentation, and empirically confirming the significant impacts of academic performance and social media environments on college students’ career choices, providing a practical paradigm for constructing multi-source data fusion career decision-making models (Yang and Chang, 2023).

Borra et al. built a machine learning classification model using Python, taking students’ talents and traits as core dimensions and designing a career path prediction logic of “strength matching-weakness exclusion,” providing an operable technical tool for college students’ independent career decision-making (Harsha et al., 2022).

#### 2.2.2 Application verification and multi-technology integration of career decision-making models

Guleria and Sood (2023) proposed a student career counseling framework integrating machine learning and explainable artificial intelligence, analyzing educational datasets containing academic and employability attributes through collaborative white-box and black-box models, effectively improving model transparency and verifying the application potential of explainable artificial intelligence in providing interpretable support for career decision-making. To mitigate biases caused by direct application of Western career theories, Qamhieh et al. (2020) developed a personalized career path recommendation system for middle school students in the Middle East, embedding regional cultural factors into machine learning model design for the first time, providing important references for localized construction of career decision-making models for college students in developing regions. Ceschi et al. (2017) posited that decision-making competence (DMC) would be considered as a construct, reflecting those behavioral characteristics related to decision-making processes fostering positive outcomes concerning the working life.

Through large-scale literature retrieval and empirical analysis, the academic community has systematically verified the application value of machine learning in career decision-making, promoting the integration of machine learning with fuzzy logic and further expanding the technical boundaries of career decision-making. Manganello et al. (2025) systematically searched the Scopus and Web of Science databases and found that existing studies mostly use supervised or hybrid machine learning techniques to develop career decision recommendation and prediction models. Although their positive roles in post-graduation education and career guidance have been verified, empirical evidence on the educational effectiveness and user impact of models remains limited. They proposed that more rigorous empirical research, higher methodological transparency, and integration of educational perspectives are needed to achieve responsible application of machine learning systems in career decision-making guidance.

To address information ambiguity in career decision-making, Cheng (2025) integrated machine learning with fuzzy logic models to realize computable transformation of fuzzy decision-making information such as “likes programming” and “good communication skills,” and combined multi-modal fusion deep learning models to capture complex relationships between personal interests, abilities, and career trends, providing a smarter and more accurate comprehensive solution for college students’ career decision-making.

## 3 Participants and methods

### 3.1 Participants

Before conducting the survey, this study collected the willingness of the research subjects to participate. Respondents were selected from higher vocational colleges in six provinces, including Jiangsu, Guangdong, Jiangxi, Anhui, Guangxi, and Guizhou, which agreed to participate in the survey. This study ultimately selected 18 higher vocational colleges as samples, and the questionnaires were distributed by online platform. All participants were informed of the purpose, intended use of the results, and filling requirements of the survey before completing the questionnaire, and agreed to allow the researchers to use the information for related research. This study promises to strictly keep all information confidential and use it solely for scientific research. A total of 3,039 questionnaires were collected. After excluding invalid questionnaires due to routine responses or insufficient completion time, 2,490 valid questionnaires were retained, with an effective response rate of 81.93%. There were 809 male students (32.5%) and 1,681 female students (67.5%). 1,457 freshmen (58.51%), 784 sophomores (31.49%), and 249 juniors (10.00%).

### 3.2 Measures

#### 3.2.1 Sense of place scale

The Sense of Place Scale developed by Fan and Li (2016) was used with minor wording revisions to fit the participants without changing item meaning. It was scored on a 5-point Likert scale (1 = strongly disagree, 5 = strongly agree), with higher total scores indicating stronger sense of place. The Cronbach’s *α* coefficient in this study was 0.896.

#### 3.2.2 Parental career-related behaviors scale

The Chinese version of the Parental Career-Related Behaviors Scale translated by Ma (2020) based on Dietrich and Kracke (2009) was adopted, including three subscales: support, interference, and neglect. It was scored on a 5-point Likert scale (1 = strongly disagree, 5 = strongly agree), with higher scores indicating stronger tendency in the dimension. The Cronbach’s *α* coefficients of the three subscales were 0.865, 0.875, and, 0.852, respectively.

#### 3.2.3 Psychological resilience scale

The Chinese version of the Connor-Davidson Resilience Scale revised by Yu and Zhang (2007) and developed by Connor and Davidson (2003) was used, including three dimensions: tenacity, strength, and optimism, with 25 items. It was scored on a 5-point Likert scale (1 = never, 5 = always), with higher total scores indicating stronger psychological resilience. The Cronbach’s *α* coefficients of the three subscales were 0.907, 0.888, and, 0.729, respectively.

#### 3.2.4 Core self-evaluations scale

The Core Self-Evaluations Scale (CSES) developed by Judge and revised by Du et al. (2012) was used, covering self-efficacy, neuroticism, and self-esteem. It was scored on a 5-point Likert scale (1 = strongly disagree, 5 = strongly agree), with five reverse-scored items. Higher scores indicate higher core self-evaluation. The Cronbach’s *α* coefficient was 0.863.

#### 3.2.5 Vocational self-concept scale

The Vocational Self-Concept Scale developed by Quint and revised by Weng (2010) was used, scored on a 5-point Likert scale (1 = strongly disagree, 5 = strongly agree), with two reverse-scored items. Higher total scores indicate clearer vocational self-concept. The Cronbach’s α coefficient was 0.765.

#### 3.2.6 Meaning in life scale

The Chinese version of the Meaning in Life Scale translated and revised by Wang et al. (2016) and developed by Steger et al. was used, including two dimensions: presence of meaning and search for meaning, with 10 items. It was scored on a 7-point Likert scale (1 = strongly disagree, 7 = strongly agree), with item 6 reverse-scored. Higher total scores indicate stronger meaning in life. The Cronbach’s *α* coefficients were 0.898, and 0.846, respectively.

#### 3.2.7 Career adapt-abilities scale short form (CAAS-SF)

The Chinese short version of the Career Adapt-Abilities Scale developed by Yu et al. (2020) was used, including four dimensions: career concern, control, curiosity, and confidence, with 12 items. It was scored on a 5-point Likert scale (1 = not strong, 5 = very strong), with higher total scores indicating stronger career adaptability. The Cronbach’s *α* coefficients of the four dimensions were 0.834, 0.798, 0.831, and 0.877, respectively.

#### 3.2.8 Future time perspective scale

The Future Time Perspective Scale developed by Song (2004) was used, including five dimensions: behavioral commitment, future efficacy, long-term goal orientation, purpose awareness, and future intention. It was scored on a 4-point Likert scale (1 = strongly disagree, 4 = strongly agree), with higher total scores indicating stronger future time perspective. The Cronbach’s *α* coefficients were 0.862, 0.923, 0.884, 0.732, and, 0.629, respectively.

#### 3.2.9 Career exploration scale

The Chinese version of the Career Exploration Scale revised by Xu (2008) based on Stumpf et al. was used, including four dimensions: environment exploration, self-exploration, purposeful-systematic exploration, and information quantity. It was scored on a 5-point Likert scale (1 = very little, 5 = very much), with higher total scores indicating more active career exploration. The Cronbach’s *α* coefficients of the four dimensions were 0.915, 0.853, 0.880 and 0.905, respectively.

#### 3.2.10 Career decision-making difficulties scale

The Career Decision-Making Difficulties Scale revised by Li (2009) was used, including three dimensions: lack of readiness, information exploration difficulties, and conflicts, with 35 items. It was scored on a 5-point Likert scale (1 = strongly disagree, 5 = strongly agree), with higher total scores indicating more severe career decision-making difficulties. The Cronbach’s α coefficients of the three subscales were 0.899, 0.902 and 0.906, respectively.

### 3.3 Data analysis methods

A analytical logic of “classification-screening” was adopted for data analysis.

#### 3.3.1 Subgroup clustering

The k-means clustering algorithm was employed to partition valid participants into distinct subgroups based on all measured indicators relevant to career decision-making difficulties. Three complementary statistical criteria—the silhouette coefficient, Davies-Bouldin index and elbow method with sum of squared errors (SSE)—were jointly applied to pinpoint the optimal cluster number. The three diagnostic indicators consistently converged on three clusters: the three-cluster solution yielded the maximum silhouette coefficient, the minimum Davies-Bouldin index, and a pronounced inflection point on the SSE curve from the elbow test. Accordingly, the full sample was ultimately sorted into three mutually exclusive cohorts.

#### 3.3.2 Key determinant identification

Cluster-derived group membership was designated as the dependent categorical variable, while the nine predictive constructs served as independent predictors: sense of place, parental career-related behaviors, psychological resilience, core self-evaluations, vocational self-concept, meaning in life, career adaptability, future time perspective, and career exploration. A random forest model was constructed to screen dominant contributing factors, with 100 decision trees preset and the Gini impurity index set as the splitting criterion for internal nodes. Ten-fold cross-validation was implemented to refine model robustness, and predictors with feature importance values exceeding 1 were retained as core contributing factors for subsequent analysis.

## 4 Results and analysis

### 4.1 Data preprocessing

Data from 2,490 valid questionnaires were analyzed for the nine predictor variables and the outcome variable (career decision-making difficulties). Descriptive statistics (mean, standard deviation) are pre-sented in Table 1. The data were normally distributed with no significant skewness or kurtosis, no missing values, and met the requirements for k-means clustering.

**Table 1:** Descriptive statistics of study variables (*N* = 2,490).

Variable	Score range	Mean	SD
Sense of place	6–30	19.73	4.71
Parental career behaviors	15–75	43.27	7.33
Psychological resilience	38–125	84.72	15.31
Core self-evaluation	10–50	32.97	6.24
Vocational self-concept	6–30	19.10	3.65
Meaning in life	15–70	50.15	9.17
Career adaptability	22–60	45.98	7.34
Future time perspective	34–100	70.83	9.77
Career exploration	18–90	60.08	12.82
Career decision-making difficulties	35–172	102.03	22.06

### 4.2 K-means clustering results

As an unsupervised learning algorithm, k-means clustering autonomously assigns observations into discrete subgroups and outperforms hierarchical clustering in computational efficiency when applied to large-scale, high-dimensional datasets. Since k-means requires pre-specified cluster quantity, three prevailing statistical indicators, namely the Silhouette Coefficient, Davies-Bouldin Index and Elbow Method, were jointly deployed to identify the optimal cluster number. Cluster solutions ranging from three to eight groups were iteratively tested across the present dataset, and all three evaluation metrics consistently pinpointed three as the optimal grouping number.

Total composite scores of the nine predictors, covering sense of place, parental career-related behaviors, psychological resilience, core self-evaluations, vocational self-concept, meaning in life, career adaptability, future time perspective and career exploration, served as clustering indicators to partition the full sample of 2,490 vocational students into three distinct subgroups and the k-means clustering applied the mean scores of the 9 predictor variables. The three clusters were labelled high-resource group, moderate-resource group and low-resource group, accounting for 18.8, 43.6 and 37.6% of total participants, respectively. Detailed clustering distribution, subgroup proportions and group-specific scores on all nine variables are summarized (Table 2).

**Table 2:** Comparisons of multi-dimensional variables among three groups (*M* ± SD).

Group	Sample (n)	Percentage (%)	Sense of place (*M* ± SD)	Parental career behaviors (*M* ± SD)	Psychological resilience (*M* ± SD)	Core self-evaluation (*M* ± SD)	Vocational self-concept (*M* ± SD)	Meaning in life (*M* ± SD)	Career adaptability (*M* ± SD)	Future time perspective (*M* ± SD)	Career exploration (*M* ± SD)
High-resource	468	18.8	23.52 ± 4.19	44.14 ± 9.16	104.57 ± 11.74	40.11 ± 5.82	23.41 ± 3.51	62.27 ± 6.53	55.78 ± 4.30	84.10 ± 7.04	75.11 ± 11.25
Medium-resource	1,085	43.6	19.75 ± 4.19	43.27 ± 6.90	86.61 ± 9.89	33.51 ± 4.45	19.38 ± 2.41	50.56 ± 6.01	47.41 ± 4.15	72.49 ± 5.36	61.73 ± 8.93
Low-resource	937	37.6	17.83 ± 3.89	42.85 ± 6.72	72.62 ± 9.67	28.79 ± 4.52	16.61 ± 2.64	43.58 ± 5.28	39.42 ± 4.52	62.26 ± 5.56	50.66 ± 8.75

Based on the conclusion of the k-means clustering algorithm, it can be concluded that there are significant differences in the level of career decision-making difficulties among different groups. The specific score results are as follows (Table 3).

**Table 3:** Comparisons of career decision-making difficulties among three groups (M ± SD).

Group	Sample (n)	Career decision-making difficulties (*M* ± SD)	Level
High-resource	468	85.26 ± 28.21	Low
Medium-resource	1,085	101.79 ± 19.09	Moderate
Low-resource	937	121.16 ± 13.30	High

### 4.3 Core determinants underlying cluster differentiation in career decision-making difficulties

Random forest, a supervised learning technique, was adopted following k-means clustering to unpack the core drivers behind subgroup divergence. Unlike unsupervised k-means clustering which only assigns participants into separate clusters without illuminating grouping rationales, random forest quantifies variable importance to identify critical predictors accounting for between-cluster discrepancies. Variable importance metrics objectively quantify which constructs dominate inter-group separation; meanwhile, the algorithm serves as a validity check for clustering outputs. Robust clustering corresponds to clear inter-cluster boundaries within the original feature space, evidenced by high predictive accuracy for cluster labels and concentrated importance values across a small subset of core variables. Additionally, random forest mitigates analytical complexity for high-dimensional datasets by screening dominant predictors and eliminating trivial indicators. The random forest model was configured with 100 decision trees, select those with a contribution degree greater than 1 as the items that play crucial roles in the clustering effect. By random forest, four variables yielded importance values above the predefined threshold of 1: future time perspective, core self-evaluations, career exploration, and psychological resilience. These four constructs are therefore identified as the dominant factors discriminating vocational students across career-indecision-based subgroups.

## 5 Mediation effect analysis

### 5.1 Future descriptive statistics and correlation analysis of study variables

All pairwise correlations among future time perspective, core self-evaluations, psychological resilience and career exploration were significantly positive, whereas each of the four variables correlated negatively with career decision-making difficulties at the 0.01 significance level (Table 4).

**Table 4:** Descriptive statistics and Pearson correlation matrix for all variables.

No.	Variable	*M*	SD	1	2	3	4	5
1	Future time perspective	3.54	0.49	–				
2	Core self-evaluation	3.30	0.62	0.63**	–			
3	Psychological resilience	3.39	0.61	0.65**	0.60**	–		
4	Career exploration	3.34	0.71	0.62**	0.45**	0.62**	–	
5	Career decision-making difficulties	2.92	0.63	−0.55**	−0.56**	−0.38**	−0.41**	–

*p < 0.05, **p < 0.01, ***p < 0.001.

### 5.2 Sequential mediation test

Hayes’ nonparametric percentile Bootstrap approach embedded in SPSS macro Model 6 was employed to test the serial mediating paths of core self-evaluations, psychological resilience and career exploration between future time perspective and career decision-making difficulties, with 5,000 resampling iterations set for bias correction.

Regression outputs revealed that future time perspective exerted significant positive predictive effects on core self-evaluations (*β* = 0.8064, *p* < 0.001), psychological resilience (*β* = 0.5674, *p* < 0.001), consistent with prior findings (Cao, 2023), and career exploration (*β* = 0.5786, *p* < 0.001), matching empirical results reported in Reference (Liu, 2022); meanwhile, it negatively predicted career decision-making difficulties (*β* = −0.4162, *p* < 0.001). Core self-evaluations positively predicted psychological resilience (*β* = 0.3086, *p* < 0.001), which aligns with the evidence from Reference (Zheng et al., 2023). Its predictive coefficient for career exploration contained zero within the corresponding confidence interval, signifying a statistically insignificant linkage, while it negatively forecasted career decision-making difficulties (*β* = −0.3866, *p* < 0.05), in line with Reference (Wu, 2015). Psychological resilience positively predicted career exploration (*β* = 0.4330, *p* < 0.001) and career decision-making difficulties (*β* = 0.1391, *p* < 0.001). Career exploration was a significant negative predictor of career decision-making difficulties (β = −0.1032, *p* < 0.001), consistent with outcomes documented across References (Wang, 2014; Fang et al., 2017; Chen et al., 2025). After all covariates were incorporated into the regression specification, the direct predictive path from future time perspective to career decision-making difficulties remained statistically meaningful (*β* = −0.4162, *p* < 0.001) (Table 5).

**Table 5:** Regression results of serial mediation model.

Regression equation		Model fit indices			Effect size & significance		95% confidence interval	
Dependent variable	Predictor variable	*R*	*R* ^2^	*F*	*β*	*t*	LICI	ULCI
Core self-evaluations	Future time perspective	0.6305	0.3976	1641.9658	0.8064	40.5212***	0.7673	0.8445
Psychological resilience	Future time perspective	0.6933	0.4806	1150.6418	0.5647	24.1889***	0.5190	0.6105
	Core self-evaluations				0.3086	16.9020***	0.2728	0.3444
Career exploration	Future time perspective	0.6834	0.4671	726.2517	0.5786	18.9277***	0.5186	0.6385
	Core self-evaluations				−0.0227	−1.1321	−0.0702	0.0188
	Psychological resilience				0.4330	18.3294***	0.3867	0.4793
Career decision-making difficulties	Future time perspective	0.6226	0.3976	393.2513	−0.4162	−13.4115***	−0.4770	−0.3553
	Core self-evaluations				−0.3866	−17.9345***	−0.4289	−0.3443
	Psychological resilience				0.1391	5.8220***	0.0922	0.1859
	Career exploration				−0.1032	−5.4215***	−0.1405	−0.0659

*p < 0.05, **p < 0.01, ***p < 0.001.

**Figure 1:**
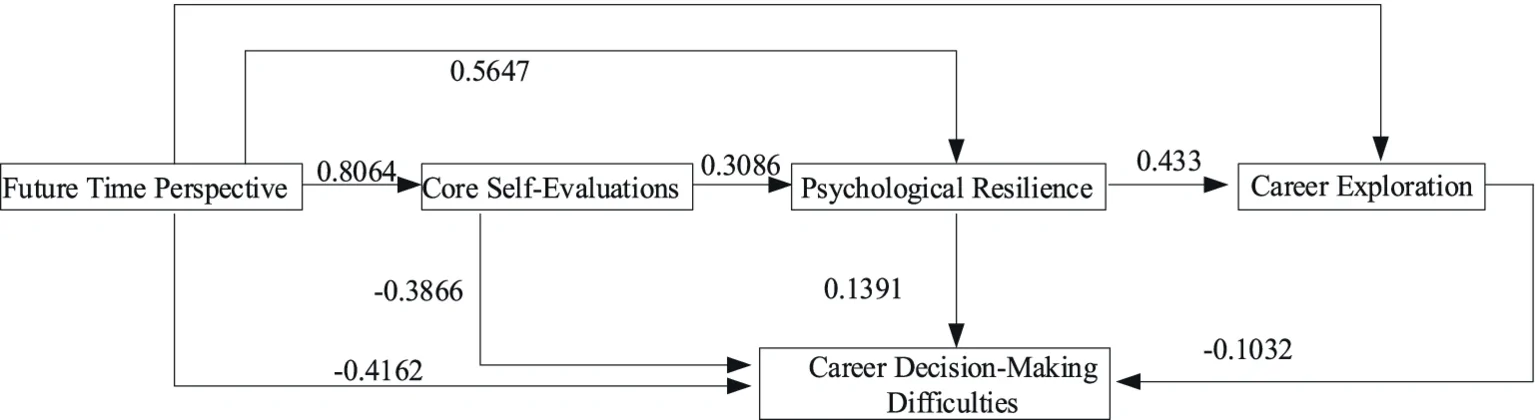
Chain mediation effect model diagram.

Mediation results (Figure 1; Table 6) indicated that future time perspective shapes career decision-making difficulties through both direct and indirect pathways, with core self-evaluations, psychological resilience and career exploration serving as partial mediators in this relational chain. Six distinct indirect paths constituted the total indirect effect:

1. Future time perspective → Core self-evaluations → Career Decision-Making Difficulties.
2. Future time perspective → Psychological resilience → Career Decision-Making Difficulties.
3. Future time perspective → Career exploration → Career Decision-Making Difficulties.
4. Future time perspective → Core self-evaluations → Psychological resilience → Career Decision-Making Difficulties.
5. Future time perspective → Psychological resilience → Career exploration → Career Decision-Making Difficulties.
6. Future time perspective → Core self-evaluations → Psychological resilience → Career exploration → Career Decision-Making Difficulties.

**Table 6:** Bootstrap results for mediation effect testing.

Path	Effect value	Bootstrap standard error	95% confidence interval
Total Effect	−0.7087	0.0216	[−0.7511, −0.6663]
Direct Effect	−0.4162	0.0310	[−0.4770, −0.3553]
Indirect Effect	−0.2925	0.03	[−0.3519, −0.2344]
FTP → SCE → CDD	−0.3117	0.0235	[−0.3519, −0.2344]
FTP → PR → CDD	0.0785	0.0177	[0.0437, 0.1145]
FTP → CE → CDD	−0.0597	0.0156	[−0.0914, −0.0300]
FTP → SCE → PR → CDD	0.0346	0.0077	[0.0196, 0.0502]
FTP → PR → CE → CDD	−0.0252	0.0068	[−0.0390, −0.0126]
FTP → SEC → PR → CE → CDD	−0.0111	0.0031	[−0.0177, −0.0053]

FTP, Future Time Perspective; CSE, Core Self-Evaluations; PR, Psychological Resilience; CE, Career Exploration; CDD, Career Decision-Making Difficulties.

As documented in Table 6, none of the 95% confidence intervals for these six indirect effects contained zero, confirming statistical significance for all six mediating pathways.

## 6 Discussion

### 6.1 Future time perspective, core self-evaluations, career exploration, and psychological resilience as core protective factors against career decision-making difficulties

Consistent with prior studies based on undergraduate samples, future time perspective, core self-evaluations, career exploration and psychological resilience all negatively predict career decision-making difficulties. Existing scholarship has validated that these resources relieve decisional hesitation among general youth populations; however, our findings reveal distinct action pathways for vocational students, which explains minor divergences from research on four-year university students. Unlike undergraduates who face diverse disciplinary career options, vocational learners are oriented toward technical posts with clear skill requirements. For them, future time perspective functions not only as a general long-term motivation but also as a guide to link daily skill training to occupational demands—this context-specific mechanism is rarely highlighted in previous research focused on liberal arts undergraduates.

One hand, the four factors jointly expand the protective factor system of career construction theory. Career construction theory emphasizes individuals’ adaptation resources when confronting vocational transitions, yet past measurements mostly overlook the layered combination of temporal cognition, self-perception, exploratory behavior and stress resistance. This study supplements a four-dimensional protective framework suited to vocational education contexts. Each factor bears an irreplaceable theoretical function: future time perspective acts as motivational antecedent, core self-evaluations as intrapsychic foundation, career exploration as behavioral mediator, and psychological resilience as stress buffer. Their independent protective effects together form a complete resource chain for resolving vocational decision struggles. On the hand, vocational colleges cannot rely on fragmented single-session career lectures. Instead, systematic training targeting the four core dimensions should be embedded into regular professional courses. For instance, instructors can design long-term career goal assignments to cultivate future time perspective, set up professional skill assessment activities to improve core self-evaluations, organize factory internships and industry surveys to boost career exploration, and deliver group psychological workshops to enhance resilience.

### 6.2 Heterogeneity in career decision-making difficulties among vocational student subgroups

The k-means cluster analysis categorized vocational college students into three mutually exclusive and stable subgroups: high-resource, moderate-resource, and low-resource groups. The three subgroups exhibited distinct and continuous gradient differences in multidimensional developmental resources and career decision-making difficulties levels, strongly verifying significant subgroup heterogeneity in vocational students’ career decision-making difficulties.

Previous career classification studies mostly divide students into “decided” and “undecided” binary groups, while our three-type gradient classification supports the perspective of conservation of resources theory: developmental resources exist in cumulative combinations rather than isolated states. The three subgroups confirm that career decision struggles stem from holistic resource deprivation rather than deficiency of a single variable. This result differs from binary classification outcomes in undergraduate research because vocational students face more concentrated employment pressure and weaker family career support on average, producing a distinct moderate-resource majority group accounting for nearly half of the sample. The heterogeneity findings deliver targeted practical implications for differentiated career intervention. The moderate-resource group, the main body of vocational learners, is suitable for standardized universal career courses and group vocational information counseling. The low-resource high-risk subgroup requires precise screening, long-term one-on-one psychological guidance and sustained family-school joint support, while high-resource students can be arranged to take part in career mentor programs as peer role models. Practically, schools are suggested to build a tiered intervention system instead of adopting unified career education for all students. Collectively, the results indicate that career decision-making difficulties is not merely a univariate high-low disparity but a typological divergence shaped by differentiated combinations of multidimensional developmental resources.

### 6.3 Predictive and explanatory power of multidimensional variables for career decision-making difficulties

In real-world career development, individual vocational decision-making relies on the synergistic effects of multiple factors rather than a single competency or supportive resource. By treating the nine variables as an integrated system for subgroup classification, this study effectively captures such synergistic combination effects. Accordingly, interventions for vocational students’ career decision-making difficulties should adopt a systematic perspective rather than focusing on single-factor improvement. Holistic optimization of psychological resources, career competencies, familial support, and place identity is essential to establish a multidimensional collaborative promotion mechanism. Vocational college students are in a critical transitional stage of career exploration and establishment, with core developmental tasks centering on self-clarification, occupational exploration, directional confirmation, and decision commitment. The prominent deficiencies of the low-resource group—including unclear self-concept, insufficient exploration, poor adaptability, and low decision confidence—reflect their failure to complete fundamental career developmental tasks. The moderate-resource group achieves basic vocational adaptation but requires strengthened goal setting and behavioral engagement to advance toward mature decision-making. In contrast, the high-resource group successfully fulfills core career exploration and decision preparation tasks. This study suggests that fundamental improvements in vocational students’ career decision-making ability and reductions in career decision-making difficulties depend on systematic intervention targeting the four core dimensions: future time perspective, core self-evaluations, career exploration, and psychological resilience. The results provide novel empirical evidence for understanding the stage-specific career developmental characteristics of vocational college students.

## 7 Implications and recommendations

### 7.1 Constructing a targeted career education curriculum system focusing on four core factors

Vocational colleges should break through the limitations of traditional employment guidance and develop a full-cycle curriculum and training system for career development, with future time perspective, core self-evaluations, career exploration, and psychological resilience as the core training dimensions. First, universities should strengthen the cultivation of students’ future time perspective through targeted exercises including future vision design, long-term goal decomposition, and career pathway planning. Such training fosters students’ long-term developmental awareness and effectively reduces short-sighted and blind career decision-making tendencies. Second, systematic professional training focusing on self-esteem, self-efficacy, internal control beliefs, and emotional stability should be designed to comprehensively improve students’ core self-evaluations and help them establish rational and positive self-perception. Third, career exploration education should be fully embedded in vocational teaching. Practical activities such as industry visits, on-the-job internships, and professional experience programs allow students to understand diverse occupational formats and career trajectories beyond classroom knowledge, enabling them to gradually clarify their personal developmental orientations. Fourth, institutions should enhance students’ psychological resilience by delivering systematic training on stress coping, emotional regulation, and frustration tolerance, so as to improve their psychological recovery capacity when facing decisional conflicts and developmental challenges. Systematic and regular cultivation of the four core competencies can fundamentally enhance vocational college students’ overall career decision-making capabilities and reduce their degree of career decision-making difficulties.

### 7.2 Implementing group-based precision career guidance according to subgroup heterogeneity

Drawing on the three typologies identified in this study—high-resource, moderate-resource, and low-resource subgroups-differentiated and precise guidance strategies should be adopted to achieve tailored intervention for each student cohort. For the high-resource group, intervention priorities focus on quality improvement and capability enhancement. Advanced career planning training, high-level practical platforms, and competitiveness promotion guidance can be provided to support their high-quality employment and long-term vocational development. For the moderate-resource group, interventions aim to compensate for developmental weaknesses. Targeted training on career adaptability, future planning, and proactive exploratory behaviors is essential to promote their transition toward the high-resource developmental level. For the low-resource group, full-process support and focused assistance are required. Priority should be given to psychological empowerment, goal activation, and basic decision-making training to improve their psychological resilience, sense of meaning in life, and planning awareness, so as to effectively alleviate severe career decision-making difficulties. Precise subgroup identification and matched personalized services can significantly improve the pertinence and effectiveness of career counselling in vocational education.

### 7.3 Establishing a full-chain career support mechanism through multi-stakeholder collaboration

To accommodate the systematic developmental needs of vocational students, this study proposes a three-dimensional supportive framework integrating psychological empowerment, practical support, and home-school collaboration. First, a comprehensive psychological support and early intervention system should be established by combining career assessment and psychological screening. This mechanism enables timely identification of students with high-risk career decision-making difficulties and delivers personalized, follow-up counselling services. Second, practical education platforms should be further optimized. Relying on school-enterprise cooperation, internship training, and vocational experience activities, vocational colleges can enrich students’ career exploration experiences, enhance their occupational cognition and information acquisition ability, and reduce decision-making dilemmas caused by information deficiency. Third, home-school collaborative mechanisms need to be strengthened. Parents should be guided to adopt scientific career-related parenting behaviors, replacing excessive interference or parental disengagement with emotional support and autonomy encouragement, so as to build a stable and positive external developmental environment. Multi-stakeholder joint efforts can comprehensively optimize the career development ecosystem for vocational students, providing solid guarantees for improving their career decision-making quality and long-term developmental outcomes.

## Data Availability

The raw data supporting the conclusions of this article will be made available by the authors, without undue reservation.
